# Plant Growth Modulates Metabolites and Biological Activities in *Retama raetam* (Forssk.) Webb

**DOI:** 10.3390/molecules23092177

**Published:** 2018-08-29

**Authors:** Mariem Saada, Hanen Falleh, Marcelo D. Catarino, Susana M. Cardoso, Riadh Ksouri

**Affiliations:** 1 Laboratory of Aromatic and Medicinal Plants, Biotechnology Center of Borj-Cédria, BP 901, Hammam-lif 2050, Tunisia; saadamariem@gmail.com (M.S.); hanenfalleh@gmail.com (H.F.); 2 Department of Chemistry & Organic Chemistry, Natural Products and Food Stuffs Research Unit (QOPNA), University of Aveiro, 3810-193 Aveiro, Portugal; mcatarino@ua.pt (M.D.C.); susanacardoso@ua.pt (S.M.C.)

**Keywords:** *Retama raetam*, physiological stage, lipophilic and hydrophilic compounds, GC-MS, LC-DAD-ESI/MS^n^, biological activities

## Abstract

This work focuses on the variability of *Retama raetam* (Forssk.) Webb bioactive compounds as a function of the plant cycle. The main results showed that it exhibited the highest percentage of polyunsaturated fatty acids, along with superior levels of vitamin C and total phenolic compounds (66.49%, 645.6 mg·100 g**^−^**^1^ FW and 23.9 mg GAE·g**^−^**^1^, respectively) at the vegetative stage. Instead, at the flowering and mature fruiting stages, *R. raetam* (Forssk.) Webb exhibited notable contents of proline (25.4 μmol·g**^−^**^1^ DW) and carotenoids (27.2 μg·g**^−^**^1^ FW), respectively. The gathered data concerning the antioxidant activity highlighted the effectiveness of the vegetative stage in comparison to the other periods. Actually, IC_50_ and EC_50_ values of the hydromethanolic extract obtained from the plant shoots at the vegetative stage were of 23, 380, 410, 1160 and 960 μg·mL^−1^ (DPPH^•^ and ABTS^•+^ radicals scavenging activity, reducing power, chelating power and β-carotene bleaching activity, respectively). Furthermore, the four studied stages showed appreciable antibacterial capacities against human pathogens with a higher efficiency of the vegetative stage extract. Finally, the LC-DAD-ESI/MS^n^ analysis revealed the predominance of isoflavonoids as main class of phenolic compounds and demonstrates that individual phenolic biosynthesis was clearly different as a function of plant growth. These findings highlight that reaching the optimum efficiency of *R. raetam* (Forssk.) Webb is closely linked to the physiological stage.

## 1. Introduction

Antioxidants, i.e., molecules that decelerate or prevent oxidation by impairing the oxidation chain reactions, can be classified in hydrophilic and hydrophobic compounds, according to their polarity [1]. In general, water-soluble antioxidants react with oxidants in the cell cytoplasm and the blood plasma, while lipid-soluble antioxidants protect cell membranes from lipid peroxidation [2]. These compounds may be biosynthesized or obtained from the diet. Notably, antioxidants have a wide range of applications, particularly as food additives and health-promoting ingredients in the formulations of functional foods and nutraceuticals, among others [3].

Plants are good sources of bioactive compounds, including lipophilic antioxidants, such as tocopherols, carotenoids, and unsaturated fatty acids, as well as hydrophilic antioxidants, including ascorbic acid and phenolic compounds. These bioactive compounds exhibit beneficial effects such as antioxidant activity, inhibition or induction of enzymes, inhibition of receptor activities, and induction and inhibition of gene expression [4,5,6].

The antioxidant levels of plants are, however, dependent on various factors, including their growth phase. According to Lisiewska et al. [7], the phenolic content in higher plants may reflect their physiological status and developmental stages. In turn, physiological stages of the plant are closely related to climatic conditions. For instance, high exposure to sunlight in summer interferes in the biosynthesis of both primary and secondary metabolites [8]. Phenolic biosynthesis is enhanced by light, and flavonoid formation is absolutely light dependent and its biosynthetic rate is related to light intensity and density [9]. In the same context, different shade levels have been shown to affect the morphological and physiological characteristics of *Hypericum perforatum*, as well as its secondary metabolite (e.g., phenolic compounds) contents [10].

**Figure 1:**
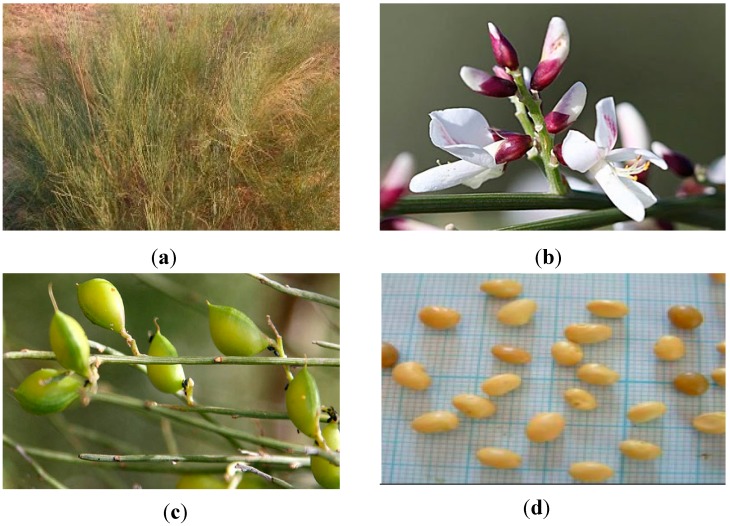
*R. raetam* (Forssk.) Webb different growth stages: aerial parts of the plant at the vegetative stage (**a**) and details from reproductive stages, namely flowers at the flowering stage (**b**), green fruits at fresh fruiting stage (**c**), and mature fruits at mature fruiting stage (**d**).

*Retama raetam* (Forssk.) Webb is a perennial shrub used in traditional medicine for the treatment of several diseases such as diabetes, hepatitis, jaundice, sore throat, skin diseases, joint pain, rheumatism, fever, inflammation, eczema, and microbial infections [11,12]. The plant is also used in Tunisia as a remedy against snake bites [13]. Research undertaken on genus *Retama* showed that aqueous extracts from *R. raetam* (Forssk.) Webb shoot exhibited diuretic and hypoglycemic effects [14]. Besides, shoot extracts reduced the concentration of triglycerides in the rats’ plasma and significantly decreased their weight [14]. Moreover, recent works showed the antimicrobial and antioxidant capacities of *Retama raetam* essential oils and its interesting potential use in the food and pharmaceutical industries [15,16]. In addition, the previous study of our group highlighted the antioxidant and antimicrobial capacities of this species [17]. To improve the exploitation of this halophyte, the aim of this work is to further understand the changeability of lipophilic (fatty acids, carotenoids) and hydrophilic (ascorbic acid, proline, and phenolic compounds) antioxidants, as well as their biological activities (antioxidant and antimicrobial), of *R. raetam* (Forssk.) Webb during its growth cycle, which include vegetative, flowering, fresh fruiting, and mature fruiting stages (Figure 1).

## 2. Results and Discussion

### 2.1. Lipophilic Compounds

The total carotenoid contents varied significantly from 2.67 to 27.19 μg·g^−1^ FW, with the minimum and maximum levels being registered in the fresh and mature fruiting stages, respectively (Table 1). Note that the superior concentration of carotenoids in mature fruits compared to immature ones has been previously reported for other plants species, e.g., Howard et al. [18] demonstrated that the content of β-carotene and β-cryptoxanthin were raised from 0.02 μg·g^−1^ FW to 3.4 μg·g^−1^ FW along pepper maturation. It is possible that the carotenoids richness in *R. raetam* (Forssk.) Webb mature fruiting stage is partly associated with the involvement of these secondary metabolites in seed dispersion and therefore plant perpetuation [19].

The fatty acid profile of *R.* *raetam* (Forssk.) Webb also varied as a function of the plant grown cycle, both qualitatively and quantitatively. Indeed, while the fat portion of mature fruit plants contained eleven fatty constituents, those of the vegetative and flowering stages were composed of eight, while only five were detected at the fresh fruiting stage. Regardless, palmitic acid (C16:0) and linoleic acid (C18:2) were major representative fatty acid at all plant stages. Additionally, α-linolenic acid (C18:3) was also a main fatty component in vegetative, flowering, and fresh fruiting stages (39–45 relative %). In turn, the mature fruiting stage was undoubtedly the most distinguishable in terms of fatty acid composition, due to the unique presence of oleic acid (C18:1), which was absent in the other physiological phases. Overall, this stage was characterized by the lowest content of saturated fatty acids (19% versus 31–40%) and a high representativeness of unsaturated fatty acids, composed of MUFA (25% versus undetected) and PUFA (54% versus 57–66%). Note that according to Richard et al. [20], there is generally a significant increase in the total content of mono-unsaturated fatty acids and a decrease in poly-unsaturated fatty acids during the plant growth until fruit ripening. Changeability in fatty acid content and composition may be related to the seasonal variation of temperature and light. Accordingly, various seaweeds from cold environments have higher degree of unsaturation compared to others from warm environments [21]. This is possibly an environmental acclimatization, since PUFAs have a lower melting point than SFAs and therefore provide a physiological advantage in cold environments by increased membrane lipid fluidity [22]. As mentioned above, the climatic conditions corresponding to *R. raetam* (Forssk.) Webb development varies from hot and sunny weather (vegetative stage) to cold and shady climate (reproductive stages), justifying the variability between the main classes of fatty acids.

**Figure 2:**
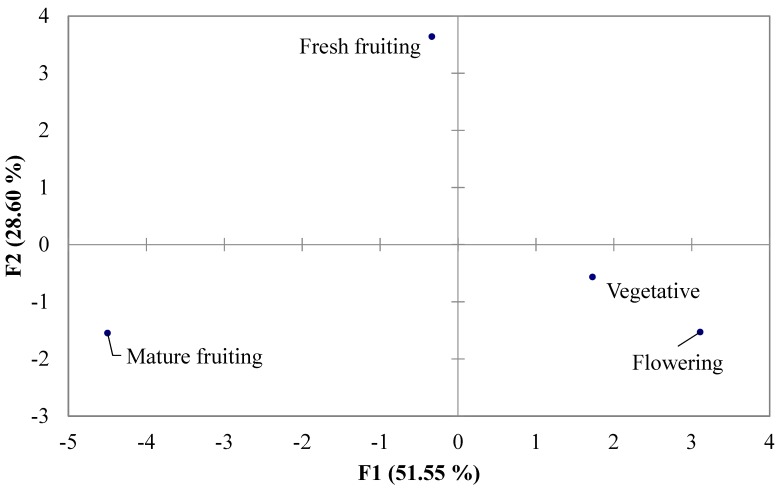
Principal components analysis of different stages based on the fatty acid composition of *Retama raetam* (Forssk.) Webb (Table 1).

Based on fatty acid composition, the results obtained from PCA (Figure 2) clearly distinguished the existence of one well-defined group represented by the vegetative and flowering stages. These two periods presented almost the same percentage in saturated (31% and 35%, respectively) and polyunsaturated fatty acids (66% and 64%, respectively). However, fresh and mature fruiting stages were distinguished from the former group, since in each of these two stages (fresh and mature fruiting stages) new fatty acids were detected, discriminating them from the other periods.

### 2.2. Hydrophylic Compounds

Ascorbic acid (vitamin C) is an abundant component of plants that acts as a major redox buffer and as a cofactor for enzymes involved in regulating photosynthesis, hormone biosynthesis, and regenerating other antioxidants [23]. Its reduced form is the active one, and the increment in the proportion of oxidized ascorbic acid at the expense of the reduced form reflects an overexploitation of the latter for ROS detoxification [24].

As shown in Table 2, the levels of total (T-AA), reduced (R-AA), and oxidized ascorbic acid (O-AA) in *R. raetam* (Forssk.) Webb varied significantly along the plant growth cycle, being particularly high at the vegetative and flowering stages. Notably, at the vegetative stage, T-AA amounted for 645.61 mg·100g^−1^ FW, from which about 73% was in the oxidized form. This can probably be ascribed to the high exposition of the plant to sunlight during this stage, since ascorbic acid accumulation represents an important protective defense against sunlight cell damage [25].

In turn, proline (i.e., amino acid that might modulate oxidative stress tolerance in plants) reached its maximum levels at the flowering period (25.4 μmol·g^−1^ DW), while its amounts decreased by about half at the vegetative and fresh fruiting periods (11–12 μmol·g^−1^ DW) and to more than 90% at the mature fruiting stage. Possibly, the abundance of this compound in the flowering stage might be associated with its relevance in the stimulation of the flowering process [26]. In this regard, Lehmann et al. [27] reported that proline induces precocious flowering and enhances the formation of axillary flower buds. In addition, proline is a major constituent of pollen, where it can represent up to 70% of total free amino acids, and nectar [27]. The highest levels of proline in the flowering stage, as compared to the mature fruiting and the vegetative periods, has also been described by Kale [28] for the medicinal plant *Microphyllus convolvulus*.

The total phenolic content of the *R. raetam* (Forssk.) Webb also varied considerably among the four growth stages. This was maximum during the vegetative phase (23.93 mg GAE·g^−1^ DW) and decreased progressively until the mature fruiting phase, reaching 15.17 mg GAE·g^−1^ DW (Table 2). Changes in phenolic compounds as a function of plant physiological stage has already been discussed by other researchers. For instance, Ksouri et al. [29] registered the highest polyphenols contents of the halophyte *Salsola kali* at the vegetative period, while minimum values were observed at the flowering stage (17 and 5 mg GAE·g^−1^ DW, respectively). In this context, one should highlight that the fruit maturation process implies an important amplification of the activity of polyphenol oxidase (PPO), resulting in a notable degradation of total polyphenols from the vegetative stage to the mature fruiting one [30]. Moreover, environmental factors can also contribute for such differences, since according to the Tunisian national institute of meteorology, during the vegetative period (June–December), *R. raetam* (Forssk.) Webb was exposed to a media of 329 insolation hours per month, while this was about half for the reproductive stages. This is consistent to the general concept that phenolic compounds play a UV-protective role in the plants [25]. In this respect, Combris et al. [31] and Toor et al. [32] reported that UV rays associated with increased solar radiation stimulated phenolic metabolism and their concomitant accumulation in the plant, especially in its aerial organs.

**Figure 3:**
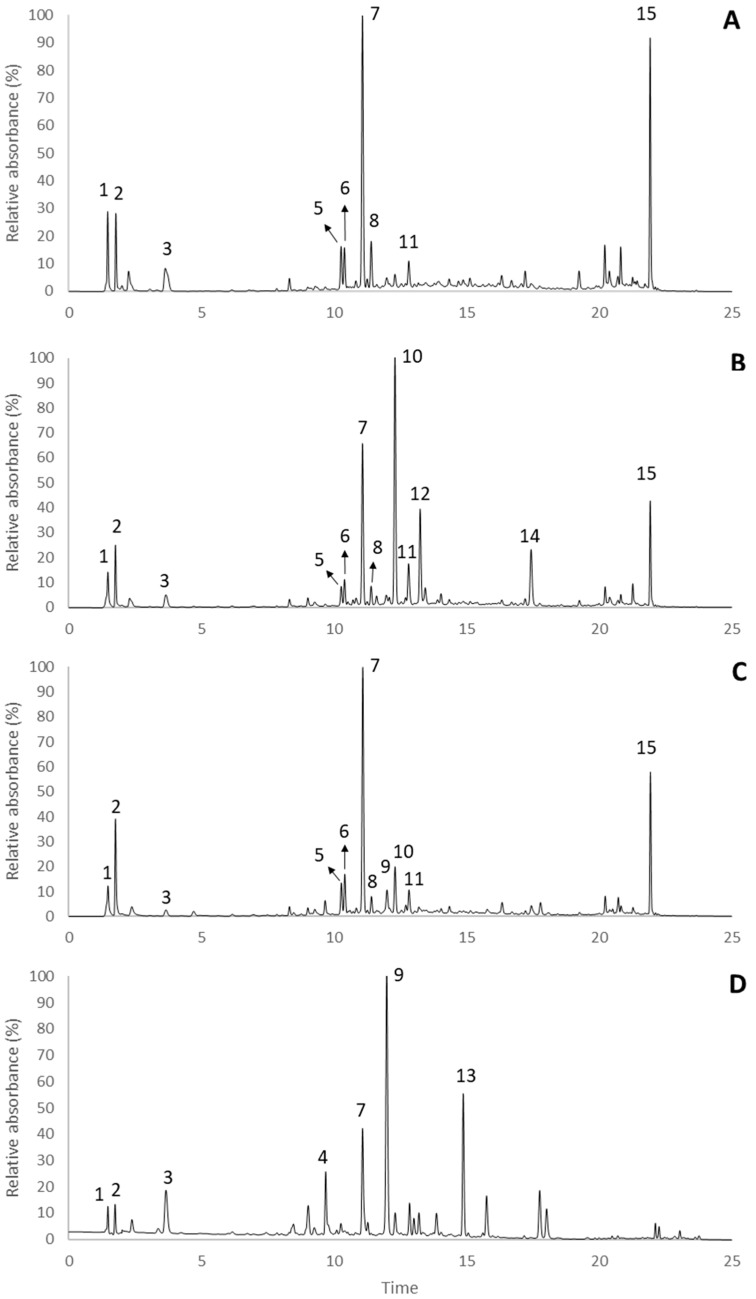
Chromatographic profiles of *R. raetam* (Forssk.) Webb hydromethanolic extracts from different seasons: (**A**) vegetative; (**B**) flowering; (**C**) fresh fruiting; and (**D**) mature fruiting stages recorded at 280 nm. Peak numbers correspond to those represented in Table 3.

In addition to the changes in the total phenolic amounts, our results also showed noticeable variations on the individual phenolic components of the plant along its growth cycle (Figure 3 and Table 3). Notably, the UHPLC-DAD-ESI/MS^n^ chromatogram of the hydromethanolic extracts obtained from *R. raetam* (Forssk.) Webb showed a high predominance of isoflavones. Overall, with exception of the extract from the mature fruiting, the phenolic profile of the extracts revealed close resemblances to each other, although great differences in peak intensities were noticeable. Among them, extracts from the vegetative and fresh fruiting stages were very similar, both being characterized by two major peaks corresponding to genistein-8-*C*-hexoside (peak **7**, λ_max_ at 261 nm, [M − H]^−^ at *m*/*z* 431), which accounted for 888.53 ± 24.57 and 628.19 ± 10.68 μg/g DW, respectively, and to an unknown compound (peak **15**, λ_max_ at 282 nm, [M − H]^−^ at *m*/*z* 311). The extract from the flowering stage showed the highest diversity of phenolic compounds, with genistein-7-*O*-glucoside, i.e., genistin (peak **10**, λ_max_ at 260 nm, [M − H]^−^ at *m*/*z* 477) becoming the most abundant compound, reaching a concentration of 867.17 ± 44.21 μg/g DW, alongside with genistein-8-*C*-hexoside that amounted for 571.74 ± 60.94 μg/g DW. Additionally, genistein (peak **14**, λ_max_ at 261 nm, [M − H]^−^ at *m*/*z* 269) and the flavone apigenin-7-*O*-glucoside (peak **12**, λ_max_ at 267 and 336 nm, [M − H]^−^ at *m*/*z* 431) also appeared as relevant compounds in this extract.

Interestingly, a clear change of the phenolic profile was noticed in the mature fruiting stage of the plant, which was characterized by the predominance of the flavanonol taxifolin (peak **9**, λ_max_ at 289 nm, [M − H]^−^ at *m*/*z* 303), the glycosylated flavone apigenin-6,8-*O*-diglucoside, i.e., vicenin 2 (peak **4**, λ_max_ at 271 and 334 nm, [M − H]^−^ at *m*/*z* 593) and a phenolic acid (peak **13**, λ_max_ at 231 and 307 nm, [M − H]^−^ at *m*/*z* 805) that was tentatively assigned to a *p*-coumaric acid derivative, since its MS fragmentation pattern showed a MS^2^ product ion at *m*/*z* 497 (equivalent to the loss of a rutinose moiety), and further MS^3^ fragments at *m*/*z* 145 (indicating a coumaroyl moiety), 351 ([M − H − 146]^−^) and 333 ([M − H − 164]^−^). Moreover, the major unknown compound detected in the remaining growth stages (peak **15**) was completely absent in the mature fruit extract. This outcome suggests that the fruit maturation process has great impact on the phenolic profile of *R. raetam* (Forssk.) Webb. Indeed, in Touati et al. [33], taxifolin was detected in considerable amounts in grains but not in the stems of *R. sphaerocarpa*, which confirms that this flavanonol is only biosynthesized in the fruits, therefore explaining its presence in the mature fruiting extracts but not in the others.

Note that previous studies showed that isoflavonoid glucosides are common in *Retama* genus [34]. In fact, isoflavones such as genistein, genistin, daidzin, daidzein, biochanin A, 6′-methoxypseudobaptegenin, and puerarin have been isolated and identified from *R. sphaerocarpa*, *R. monosperma*, and *R. raetam* [34,35]. Concerning *R. raetam*, previous phytochemical studies resulted in the isolation of a number of compounds, with the most characteristic corresponding to flavonoids and alkaloids. Flavonoids such as daidzein, vicenin-2, naringenin, apigenin, kaempferol, quercetin, and kaempferol-7-*O*-glucoside were found in the seeds [36], while daidzein 7,4′-dimethyl ether, chrysoeriol 7-*O*-glucoside, and orientin were isolated from the leaves [36]. In addition, Kassem et al. [36] described the occurrence of two new flavonoids in the aerial parts of the plant, namely luteolin 4′-*O*-neohesperidoside and 5,4′-dihydroxy-(3″,4″-dihydro-3″,4″-dihydroxy)-2″,2″-dimethylpyrano-(5″,6″:7,8)-flavone. Still, to the best of our knowledge, several of the genistein derivatives herein identified, namely genistein-*C*-hexoside-*O*-pentoside (peak **6**, λ_max_ at 261 nm, [M − H]^−^ at *m*/*z* 563), genistein-8-*C*-hexoside (peak **7**) and genistein-3-hydroxy-3-methylglutaroyl (peak **11**, λ_max_ at 262 nm, [M − H]^−^ at *m*/*z* 575) have never been reported in the genus *Retama* before, as well as tectorigenin-8-*C*-hexoside (peak **8**, λ_max_ at 262 nm, [M − H]^−^ at *m*/*z* 461), apigenin-7-*O*-glucoside (peak **12**). Compounds such as piscidic acid (peak **3**, λ_max_ at 223 and 275 nm, [M − H]^−^ at *m*/*z* 255), vicenin 2 (peak **4**), calycosin-*O*-hexoside (peak **5**, λ_max_ at 255 nm, [M − H]^−^ at *m*/*z* 491), and genistin (peak **10**) are also being reported for the first time in this species.

### 2.3. Antioxidant Activities

In this study, the antioxidant ability of the hydromethanolic extracts from *R. raetam* (Forssk.) Webb at the four growth stages was screened through in vitro methods, namely total antioxidant activity (TAA)**,** DPPH^•^ ABTS^•+^, FRAP, chelating power, and β-carotene bleaching, in order to evaluate their scavenging ability towards distinct radicals, as well as the ability to reduce Fe^3+^ to Fe^2+^, to chelate Fe^2+^, and to inhibit the bleaching of the antioxidant pigment β-carotene, respectively.

As shown in Table 4, with few exceptions, the antioxidant potency of the extracts could be generalized as vegetative stage > mature fruiting stage > fresh fruiting stage > flowering stage. The dominance of the vegetative extract regarding the remaining ones varied among the assays and was particularly evident in chelating power and β-carotene bleaching tests (IC_50_ values of mature fruiting and flowering/fresh fruiting were about 3–6.6 and 8–11 fold those found for vegetative stage). Note that the superior antioxidant potency of the hydromethanolic extract from vegetative stage, together with its richness in TPC, suggests that phenolic compounds might be responsible for such activity. Yet, the lower antioxidant capacity of the flowering/fresh fruiting stages (with TPC levels of 20.75 ± 0.02 and 18.23 ± 0.02 mg GAE·g^−1^ DW, respectively) as compared to that of mature fruiting stage (with TPC levels of 15.17 ± 0.02 mg GAE·g^−1^ DW) also suggest that non-phenolic components might also play a relevant role in the involved reactions. Interestingly, other authors also highlighted the antioxidant activity of plants in vegetative growth stages, as compared to other physiological periods. E.g., the total antioxidant activity of the halophyte *Cakile maritima* at the vegetative stage was reported by Meot-Duros et al. [37], while Ksouri et al. [29] showed the same tendency when evaluating DPPH^•^ scavenging activity of *Salsola kali* shoots.

### 2.4. Antimicrobial Activity of Shoot Extracts

The hydromethanolic extracts of *R. raetam* (Forssk.) Webb at the four growth stages displayed distinct antimicrobial effects towards the 14 tested pathogenic bacteria strains (Table 5). The most interesting antibacterial activity was provided by the extract from the vegetative stage. This was particularly active against *Bacillus cereus* and *Vibrio vulnificus* (inhibition zone diameter of 12 and 11 mm, respectively) but also had strong activity against the Gram-positive *Listeria monocytogenes,* *Enterococcus faecalis*, and *Micrococcus luteus*, as well as towards the Gram-negative *Aeromonas hydrophila, Vibrio vulnificus, Vibrio alginolyticus*, and *Vibrio cholerae* (diameter of inhibition zone between 7 and 11 mm). In turn, no activity was noted against *Staphylococcus epidermidis*, *Escherichia coli*, *Pseudomonas aeruginosa*, *Salmonella typhimurium*, *Staphylococcus aureus*, and *Vibrio parahaemolyticus*.

In general, the extract from the flowering stage also showed strong activity against the same strains (diameter of inhibition zone in the range of 7 and 12 mm), while those of fresh and/or mature fruiting failed to inhibit *Listeria monocytogenes*, *Enterococcus faecalis Vibrio vulnificus*, and *Vibrio cholerae*. The higher activity of extracts from the vegetative and flowering stages could be ascribed to their phenolic compound richness, especially flavonoids (isoflavonoids), possibly due to their adsorption to cell membranes provoking their destabilization and/or interaction with some enzymes, substrates, or metal ion deprivation, as previously suggested [38,39,40].

## 3. Materials and Methods

### 3.1. Plant Sampling and Extract Preparation

*Retama raetam* (Forssk.) Webb samples were collected from the Sebkha of Soliman (30 km from capital of Tunis; 36°42′50″ N and 10°24′31″ E; superior semi-arid bioclimatic stage; mean annual rainfall: 500–600 mm) at different periods:

1- ***Vegetative stage***: the plants, of about 2 m high, were collected in September 2010. The shrub had many long and velvety green twigs, jagged and covered with small silky white hairs (Figure 1a).
2- ***Flowering stage***: the plants, of about 2 m high, were collected in February 2011. Their flowers (Figure 1b) were white, tiny and formed by 5 to 10 petals.
3- ***Fresh fruiting stage***: the plants, of about 2 m high, were collected in April 2011. The green fruit (Figure 1c) was a small egg-shaped pod that ended with a beak.
4- ***Mature fruiting stage***: the plants, of about 2 m high, were collected in collected in May 2011. The matured fruit (Figure 1d) contained only one kidney-shaped seed of a yellow ocher.

Plants were identified by the botanist of the Biotechnology Center of Borj-Cedria (CBBC), and a voucher specimen (F-RE 27) was deposited at the Herbarium of the Laboratory of Extremophile Plants at CBBC.

Carotenoids and vitamin C analysis were performed on the shoots of fresh plants, which were kept at −80 °C until the analysis. In turn, the remaining analyses were performed with air-dried shoots (two weeks followed by oven-dried for 1 h at 40 °C).

### 3.2. Chemical Reagents

Folin–Ciocalteu reagent, sodium carbonate anhydrous (Na_2_CO_3_), gallic acid, sodium nitrite (NaNO_2_), aluminum chloride hexahydrate (AlCl_3_, 6H_2_O), 2,2-diphenyl-1-picrylhydrazyl (DPPH^•^), ninhydrin, sodium hydroxide de (NaOH), trichloroacetic acid iron, 2,2′-pipyridyl; chloride anhydrous (FeCl_3_) and catechin were purchased from Fluka (Buchs, Switzerland). ABTS single reagent, β-Carotene, Tween 40, linoleic acid, *N*-ethylmaleimide (NEM), dithiothreitol (DTT), iron dichloride (FeCl_2_)_,_ and butylated hydroxytoluene (BHT) were purchased from Sigma-Aldrich (GmbH, Sternheim, Germany). Sulfuric acid (H_2_SO_4_), potassium ferricyanide K_3_Fe(CN)_6_, glacial acetic acid, sulfosalicylic acid, ferrozine and Muller Hinton medium were purchased from Merck (Darmstadt, Germany). The phenolic standards ginestin and quercetin-7-*O*-galactoside were purchased from Extrasynthese (Genay, France).

### 3.3. Assessment of Lipophilic Compounds

#### 3.3.1. Carotenoids

Carotenoid content was determined according to the method of Nonier et al. [41]. Two mL of 80% acetone were added to samples cut into discs (200 mg each). The extraction took place in dark at 4 °C for 72 h, followed by absorbance measurement at 470, 663, and 647 nm. The total amount of carotenoids was then calculated through the Formula (1):

Carotenoids (μg·mL^−1^) = 5 × A _(470)_ + 2.846 × A _(663)_ − 14.876 × A_(647)_ (1)

#### 3.3.2. Fatty Acids

The fatty acids profile was determined by GC/MS (Gas chromatography/mass spectrometer detector), after Soxhlet extraction of 30 g of dry shoots with hexane for 4 h, followed by esterification of fat according to the previous procedure of Megdiche-Ksouri et al. [42]. In more detail, 0.2 mL of the lipidic extract was saponified with 3 mL of a methanolic sodium hydroxide solution (0.5 M) for 15 min in a water bath at 60 °C. The mixture was then homogenized with 3 mL of a methanolic solution of BF_3_ (14%) and the reaction was allowed to proceed for 5 min. Subsequently, 2 mL of water were added to the mixture and fatty acids methyl esters (FAMEs) were extracted twice with 10 mL of petroleum ether. The GC apparatus consisted of a HP-5980 Series II instrument, equipped with HP-5MS capillary column (30 m × 0.25 mm; 0.25 μm film thickness), split/splitless injector (220 °C). The oven temperature was held at 150 °C, then programmed at 15 °C/min up to 220 °C, and held isothermally at 220 °C for 5 min. Helium was the carrier gas at an initial flow rate of 1 mL/min. Split ratio was 20:1. Injection volume was equal to 2 μL FAMES components were identified by comparing their relative retention times and mass spectra with the data from the library Wiley, Mass-Finder, and Adams GC/MS libraries and their abundance and the results expressed in relative percentage of each fatty acid.

### 3.4. Assessment of Hydrophilic Compounds

#### 3.4.1. Vitamin C

The assay used in the present study is based on the reduction of Fe^3+^ to Fe^2+^ by ascorbic acid under acidic conditions [43]. The Fe^2+^ forms complexes with 2,2′-pipyridyl at 4%, producing a pink color that absorbs at 525 nm. Oxidized vitamin C was converted to reduced vitamin C by pre-incubation of the sample with dithiothreitol (DTT, 10 mM). The excess of DTT was then removed with *N*-ethylmaleimide (NEM) and the reduced Vitamin C was determined. The amount of oxidized Vitamin C was then calculated between total and reduced acid ascorbic.

#### 3.4.2. Proline

Proline was determined following the ninhydrin method described by Bates et al. [44], using L-proline as a standard. Ten mg of shoot samples were homogenized in 1.5 mL of 3% aqueous sulfosalicylic acid and centrifuged for 30 min at 14,000× *g*. To the supernatant (1 mL), 1 mL of acid ninhydrin and 1 mL of glacial acetic acid were added, and the mixture was boiled for 1 h. After extraction with 2 mL of toluene, the free proline molecule was quantified at 520 nm.

#### 3.4.3. Phenolic Compounds

Total phenolic compounds and total flavonoids were measured according to the method of Dewanto et al. [45] and the results were expressed as mg of gallic acid or mg catechin per gram of dry weight (mg CE·g^−1^ DW), respectively. In addition, individual phenolic compounds were identified by UHPLC-DAD-ESI/MS^n^ of hydromethanolic extracts (2.5 g dry powder in 25 mL of 80% methanol, for 30 min), as described elsewhere [46]. The work was carried out in Ultimate 3000 (Dionex Co., San Jose, CA, USA) apparatus with an ultimate 3000 Diode Array Detector (Dionex Co., San Jose, CA, USA) coupled to a Thermo LTQ XL (Thermo Scientific, San Jose, CA, USA) ion trap mass spectrometer equipped with an ESI source. Analysis was performed on a Hypersil Gold (Thermo Scientific, San Jose, CA, USA) C18 column (100 mm length; 2.1 mm i.d.; 1.9 μm particle diameter, end-capped) and its temperature was maintained at 30 °C. The mobile phase for the separation of *Retama raetam* (Forssk.) Webb extracts constituents was composed of (A) acetonitrile and (B) 0.1% of formic acid (*v*/*v*). The solvent gradient started with 5–40% of solvent (A) over 14.72 min, from 40–100% over 1.91 min, remaining at 100% for 2.19 more min before returning to the initial conditions. The flow rate was 0.2 mL min^−1^ and UV–Vis spectral data for all peaks were accumulated in the range of 200–500 nm while the chromatographic profiles were recorded at 280 and 340 nm. Control and data acquisition of MS were carried out with the Thermo Xcalibur Qual Browser data system (Thermo Scientific, San Jose, CA, USA). Nitrogen above 99% purity was used and the gas pressure was 520 kPa (75 psi). The instrument was operated in negative-ion mode with the ESI needle voltage set at 5.00 kV and an ESI capillary temperature of 275 °C. The full scan covered the mass range from *m*/*z* 100 to 2000. CID–MS/MS and MS^n^ experiments were simultaneously performed for precursor ions using helium as the collision gas with a collision energy of 25–35 arbitrary units.

Quantification of major compounds of each extract was also carried out and for that, calibration curves were obtained by the injection of known concentrations of different standard compounds, namely ginestin (y = 11699.79x + 33085.50; R^2^ = 0.999), quercetin-7-*O*-galactoside (y = 10138.87x – 12806.00; R^2^ = 0.995). Following a frequently adopted approach [47,48], when phenolic reference compounds were not available, the quantification was based on structurally related substances, and the results for each target phenolic compound were expressed in equivalents of the corresponding reference.

### 3.5. Evaluation of Antioxidant Activities

The antioxidant activity of *R. raetam* (Forssk.) Webb at the four seasonal stages were evaluated by distinct in vitro methods, as described in bellow. For that, extracts were obtained by magnetic stirring of 2.5 g dry powder in 25 mL 80% methanol for 30 min. The mixtures were filtered through Whatman No. 4 filter paper and maintained at 4 °C until analysis [49].

#### 3.5.1. Total Antioxidant Capacity

This assay is based on the reduction of Mo(VI) to Mo(V) by the extract and subsequent formation of a green phosphate/Mo(V) complex at acid pH [50]. An aliquot (0.1 mL) of extracts was combined to 1 mL of reagent solution (0.6 M sulfuric acid, 28 mM sodium phosphate and 4 mM ammonium molybdate). The tubes were incubated at 95 °C for 90 min. After that, the mixture was cooled to room temperature and the absorbance of each solution was measured at 695 nm against a blank. The antioxidant capacity was expressed as mg gallic acid equivalent per gram dry weight (mg GAE·g^−1^ DW).

#### 3.5.2. DPPH^•^ Scavenging Ability

The radical scavenging activity of the extracts was measured using the DPPH^•^ (1′1-diphenyl-2-picrylhydrazyl) method according to Hanato et al. [51]. For this, 1 mL of the extracts at various concentrations was mixed with 0.25 mL of a DPPH^•^-methanolic solution (0.2 mM) and allowed to react in the dark for 30 min. Then, the absorbance of the resulting solution was read at 517 nm. The antiradical activity was determined using Equation (2):

Inhibition (%) = [(A_0_ − A_1_)/A_0_] × 100 (2)

where A_0_ is the absorbance of the control at 30 min, and A_1_ is the absorbance of the sample at 30 min. The antiradical activity was expressed as IC_50_ (mg·mL^−1^).

#### 3.5.3. ABTS^•+^ Scavenging Activity

ABTS^•+^ was produced by the reaction between 5 mL of 7 mM ABTS solution and 5 mL of 2.45 mM potassium persulfate solution, stored in the dark for 16 h. Before usage, this solution was diluted with ethanol to get an absorbance of 0.700 ± 0.020 at 734 nm. The reaction mixture comprised 950 μL of ABTS^•+^ solution and 50 μL of each sample at various concentrations. The mixture was homogenized and its absorbance was recorded after 6 min at 734 nm [52]. ABTS^•+^ scavenging ability was expressed as IC_50_ (mg·mL^−1^).

#### 3.5.4. Reducing Power

The ability of the extracts to reduce Fe^3+^ was assayed by the method of Oyaizu [53]. Extracts (1 mL) were mixed with 2.5 mL of phosphate buffer (0.2 M, pH 6.6) and 2.5 mL of K_3_Fe(CN)_6_ (1%). After incubation at 50 °C for 20 min, 2.5 mL of trichloroacetic acid (10%) was added and the mixture was centrifuged at 650× *g* for 10 min. Finally, 2.5 mL of the upper layer was mixed with 2.5 mL of distilled water and 0.5 mL of aqueous FeCl_3_ (0.1%), then absorbance was measured at 700 nm. Results were expressed as EC_50_ value (mg/mL) which is the effective concentration giving an absorbance of 0.5 and was obtained from linear regression analysis.

#### 3.5.5. Ferrous Ion Chelating Activity

This activity was assessed as described by Zhao et al. [54]. Different concentrations of extracts were added to 0.05 mL of FeCl_2_, 4H_2_O solution (2 mM) and left for incubation at room temperature for 5 min. Then, the reaction was initiated by adding 0.1 mL of ferrozine (5 mM), and the mixture was adjusted to 3 mL with distilled water, shaken vigorously, and left at room temperature for 10 min. Absorbance of the solution was then measured at 562 nm. The inhibition of ferrozine-Fe^2+^ complex formation was calculated using Equation (1). Results are expressed as EC_50_, i.e., the efficient concentration corresponding to 50% ferrous iron chelating.

#### 3.5.6. β-Carotene Bleaching Test

A modification of the method described by Koleva et al. [55] was employed. β-Carotene (2 mg) was dissolved in 20 mL chloroform and to 4 mL of this solution, linoleic acid (40 mg) and Tween 40 (400 mg) were added. Chloroform was evaporated under vacuum at 40 °C and 100 mL of oxygenated ultra-pure water was added, then the emulsion was vigorously shaken. An aliquot (150 μL) of the β-carotene/linoleic acid emulsion was distributed in each of the wells of 96-well plate and solutions of the test samples (10 μL) were added. Three replicates were prepared for each sample. The plates were incubated at 50 °C for 120 min. Absorbance was measured immediately (t = 0 min) and after incubation (t = 120 min) using a model EAR 400 plate reader (Labsystems Multiskan MS) at 470 nm. The β-carotene bleaching ability of the extracts was determined using the Equation (3):

Inhibition (%) = [(As(120) − Ac(120))/(Ac(0) − Ac(120))] × 100(3)

where Ac(0) and Ac(120) are the absorbance values of the control at 0 and 120 min, respectively, and As(120) is the sample absorbance at 120 min. The results were expressed as IC_50_ values (mg·mL^−1^).

### 3.6. Evaluation of Antibacterial Activity

The antimicrobial activity of *R. raetam* (Forssk.) Webb hydromethanolic extracts (2.5 g dry powder in 25 mL 80% methanol, 30 min) at different grown stages was assessed by the agar disk diffusion method [56] against fourteen human pathogenic bacteria: Gram-positive cocci including *Micrococcus luteus* NCIMB 8166, *Enterococcus faecalis* ATCC 29212, *Listeria monocytogenes* ATCC 19115, *Staphylococcus epidermidis* CIP 106510, *Bacillus cereus* ATCC 14579, *Staphylococcus aureus* ATCC 25923, and Gram-negative bacteria including *Aeromonas hydrophila* ATCC 7566, *Vibrio parahaemolyticus* ATCC 17802, *Vibrio cholerae* non-O_1_ IPT, *Vibrio alginolyticus* ATCC 33787, *Vibrio vulnificus* ATCC 27962T, *Salmonella typhimurium* ATCC 1408, *Pseudomonas aeruginosa* ATCC 27853, *Escherichia coli* ATCC 85218. The bacterial strains were first grown on Muller Hinton medium at 37 °C for 24 h prior to seeding onto the nutrient agar. One or several colonies of the indicator bacteria were transferred into API suspension medium (BioMérieux) and adjusted to the 0.5 McFarland turbidity standard with a Densimat (BioMérieux). A sterile filter disc with 6 mm diameter (Whatman paper No 3) was placed on the infusion agar seeded with bacteria, and 10 μL per disc of the extract (300 mg/mL) was added. The treated Petri dishes were incubated at 37 °C for 24 h. The antimicrobial activity was determined by measuring the zone of growth inhibition surrounding the discs.

### 3.7. Statistical Analysis

For all plant parameters, three replicates were used. To determine the relative importance of grown stage on antioxidant compounds and biological activities, a two-way analysis of variance (ANOVA) was achieved for whole data, using the SAS system (1990) software, Version 6 (SAS Institute Inc., Cory, NJ, USA). Means were compared using the Duncan’s multiple range test at the *p* < 0.05 level, when significant differences were found. A principal component analysis (PCA) was performed to discriminate between different maturity stages on the basis of their fatty acid and phenolic composition. All analyses were performed by the “Statistica v 5.1” software [57].

## 4. Conclusions

The chemical composition, the phenolic profile, the antioxidant and antimicrobial activities of *R. raetam* (Forssk.) Webb during its growth cycle are reported. Based on our results, it is possible to infer that among the four studied stages, the vegetative one presented an interesting richness in vitamin C, polyunsaturated fatty acids, and polyphenols, consistent with the highest antioxidant and antimicrobial capacities. Thus, climatic conditions like high temperatures and light intensity found in summer and autumn (vegetative period) promote production of phenolic compounds, which is related to the high antioxidant activities throughout the development cycle. Therefore, the advised harvest time of *R. raetam* (Forssk.) Webb could be in the vegetative stage rather than the reproductive ones. Finally, these findings support the utilization of this plant in a large field of application including cosmetic, pharmaceutical, agro-alimentary, and biological defense.

## Figures and Tables

**Table 1:** Carotenoids (μg·g^−1^ FW) and fatty acid composition (relative %) in shoots of *Retama raetam* (Forssk.) Webb at different grown stages.

	Vegetative Stage	Flowering Stage	Fresh Fruiting Stage	Mature Fruiting Stage
**Carotenoids (μg·g^−1^ FW)**	16.67 ± 0.02 ^b^	9.72 ± 0.03 ^c^	2.67 ± 0.02 ^d^	27.19 ± 0.06 ^a^
**Fatty acids (relative %)**				
***Saturated***				
Lauric acid C12:0	3.68 ± 0.18 ^a^	2.36 ± 0.12 ^b^	-	0.04 ± 0.00 ^c^
Myristic acid C14:0	1.51 ± 0.07 ^a^	1.13 ± 0.05 ^a^	-	0.16 ± 0.01 ^b^
Pentadecylic acid C15:0	-	-	25.38 ± 1.27 ^a^	0.08 ± 0.00 ^b^
Palmitic acid C16:0	19.10 ± 0.95 ^b^	20.52 ± 1.02 ^a^	12.2 ± 0.61 ^c^	12.23 ± 0.61 ^c^
Margaric acid C17:0	0.52 ± 0.02 ^b^	-	-	1.28 ± 0.06 ^a^
Stearic acid C18:0	3.66 ± 0.18 ^b^	4.63 ± 0.23 ^a^	2.39 ± 0.12 ^c^	4.14 ± 0.11 ^a^
Arachidic acid C20:0	-	6.10 ± 0.30 ^a^	-	0.5 ± 0.21 ^b^
Heneicosanoic acid C21:0	-	1 ± 0.05	-	-
Behenic acid C22:0	2.24 ± 0.11 ^a^	-	-	0.18 ± 0.01 ^b^
***Unsaturated***				
Oleic acid C18 :1	-	-	-	24.97 ± 1.25
Linoleic acid C18:2	22.72 ± 1.13 ^b^	18.80 ± 0.94 ^c^	18.75 ± 0.94 ^c^	49.85 ± 2.49 ^a^
α-linolenic acid C18:3	43.77 ± 2.18 ^b^	45.37 ± 2.26 ^a^	38.67 ± 1.93 ^c^	3.99 ± 0.19 ^d^
***SFA***	30.71 ± 1.53 ^c^	34.74 ±1.74 ^b^	39.97 ± 1.99 ^a^	18.61 ± 0.93 ^d^
***MUFA***	-	-	-	24 .97 ±1.25
***PUFA***	66.49 ± 3.32 ^a^	64.17 ± 3.02 ^b^	57.42 ± 2.87 ^c^	53.84 ± 2.69 ^d^

SFA—saturated fatty acids; MUFA—monounsaturated fatty acids; PUFA—polyunsaturated fatty acids. Mean values ± S.D.; Statistical analysis was performed by one-way ANOVA, followed by Duncan test. In each line different letters mean significant differences (*p* < 0.05).

**Table 2:** Contents of ascorbic acid, proline, and total phenolic compounds (TPC) of *Retama raetam* (Forssk.) Webb shoots at different grown stages.

	Vegetative Stage	Flowering Stage	Fresh Fruiting Stage	Mature Fruiting Stage
**Vitamin C (mg·100g^−1^ FW)**				
**Total AA**	645.61 ± 0.15 ^a^	627 ± 0.18 ^b^	367 ± 0.17 ^c^	103.2 ± 0.00 ^d^
**Reduced AA**	173.2 ± 0.01 ^b^	184.5 ± 0.01 ^a^	130.6 ± 0.03 ^c^	101.2 ± 0.00 ^d^
**Oxidized AA**	472.45 ± 0.01 ^a^	442.51 ± 0.05 ^b^	236.27 ± 0.07 ^c^	2.01 ± 0.06 ^d^
**Proline (μmol·g^−1^ DW)**	12.1 ± 0.03 ^b^	25.4 ± 0.01 ^a^	11.23 ± 0.01 ^c^	1.56 ± 0.01 ^d^
**TPC (mg GAE** **·g^−1^ DW)**	23.93 ± 0.03 ^a^	20.75 ± 0.02 ^b^	18.23 ± 0.02 ^b^	15.17 ± 0.02 ^c^

Mean values ± S.D.; Statistical analysis was performed by one-way ANOVA, followed by Duncan test. In each line, different letters mean significant differences (*p* < 0.05).

**Table 3:** Identification and quantification of the main compounds from the four *R. raetam* (Forssk.) Webb stages by UHPLC-DAD-ESI/MS^n^.

Peak	RT (min)	λ_max_ (nm)	[M − H]^−^ (*m*/*z*)	ESI-MS^n^ Fragments	Proposed Compounds	Mean Content (μg/g Dry Plant Material)			
						Vegetative Stage	Flowering Stage	Fresh Fruiting Stage	Mature Fruiting Stage
**1**	1.45	303	133	MS^2^[133]: **115**	Malic acid	+	+	+	+
**2**	1.74	227, 304	191	MS^2^[191]: **111**,173	Citric acid	+	+	+	+
**3**	3.63	223, 275	255	MS^2^[255]: **165**, 193, 179, 149	Piscidic acid	+	+	+	+
**4**	9.68	271, 334	593	MS^2^[593]: **473**, 503, 353, 383, 575	Vicenin 2	ND	ND	tc	+
**5**	10.26	255	491*	MS^2^[491]: **283**, 445; MS^3^[283]: **268**	Calycosin-*O*-hexoside	+	+	+	ND
**6**	10. 39	261	563	MS^2^[563]: **311**, 283, 341, 269	Genistein-*C*-hexoside-*O*-pentoside	+	+	+	ND
**7**	11.07	261	431	MS^2^[431]: **311**, 269	Genistein-8-*C*-hexoside	888.53 ± 24.57 ^a^	571.74 ± 60.94 ^c^	628.19 ± 10.68 ^b^	+
**8**	11.93	262	461	MS^2^[461]: **341**, 371	Tectorigenin-8-*C*-hexoside	+	+	+	ND
**9**	11.98	289	303	MS^2^[303]: **285**, 177, 125; MS^3^[285]: **241**, 175, 257, 199, 217	Taxifolin	ND	ND	+	224.92 ± 4.03
**10**	12.30	260, sh325	477*	MS^2^[477]: **269**, 311	Genistin	tc	867.17 ± 44.21	+	ND
**11**	12.82	262, sh324	575	MS^2^[575]: **431**, 311, 341	Genistein-3-hydroxy-3-methylglutaroyl	+	+	+	tc
**12**	13.24	267, 336	431	MS^2^[431]: **269**	Apigenin-7-*O*-glucoside	ND	+	ND	ND
**13**	14.87	231, 307	805	MS^2^[805]: **497**, 351; MS^3^[497]: **145**, 351, 333	*p*-Coumaric acid derivative	ND	ND	ND	+
**14**	17.43	261, sh331	269	MS^2^[269]: **269**, 225, 241	Genistein	ND	+	ND	ND
**15**	22.40	282	311	MS^2^[311]: **249**, 293	Unkown	+	+	+	ND

tc, traces; ND, not detected; +, detected but not quantified; *, detected as [M − H + HCOOH]^−^ adducts. Mean values ± S.D.; Statistical analysis was performed by one-way ANOVA, followed by Duncan test. In each line different letters mean significant differences (*p* < 0.05).

**Table 4:** Total antioxidant activity (TAA), DPPH^•^ test, ABTS^•^^+^ assay, reducing power, chelating power and β-carotene bleaching activity) of the hydromethanolic extracts from *Retama raetam* (Forssk.) Webb shoots of different grown stages.

	Vegetative Stage	Flowering Stage	Fresh Fruiting Stage	Mature Fruiting Stage
**TAA (mg GAE·g^−1^ DW)**	55.6 ± 0.04 ^a^	32.3 ± 0.02 ^c^	26.02 ± 0.02 ^d^	43.73 ± 0.03 ^b^
**DPPH^•^ (IC_50_ μg·mL^−1^)**	23 ± 0.01 ^a^	160 ± 0.01 ^d^	68 ± 0.00 ^c^	20.5 ± 0.00 ^b^
**ABTS^•^** **^+^ assay (IC_50_ μg·mL^−1^)**	380 ± 0.02 ^a^	940 ± 0.01 ^d^	780 ± 0.00 ^c^	540 ± 0.01 ^b^
**Reducing power (EC_50_ μg·mL^−1^)**	410 ± 0.00 ^a^	2500 ± 0.01 ^d^	2300 ± 0.01 ^c^	805 ± 0.00 ^b^
**Chelating power (EC_50_ μg·mL^−1^)**	1160 ± 0.01 ^a^	12,292 ± 0.03 ^d^	11,933 ± 0.00 ^c^	7600 ± 0.02 ^b^
***β*-carotene bleaching (IC_50_ μg·mL^−1^)**	960 ± 0.01 ^a^	8500 ± 0.00 ^d^	7700 ± 0.00 ^c^	3100 ± 0.00 ^b^

Mean values ± S.D.; Statistical analysis was performed by one-way ANOVA, followed by Duncan test. In each line different letters mean significant differences (*p* < 0.05).

**Table 5:** Antibacterial activity of *Retama raetam* (Forssk.) Webb shoots (at 100 mg·mL**^−1^**) at different grown stages against fourteen human pathogenic bacteria.

Bacterial Strains		Vegetative Stage	Flowering Stage	Fresh Fruiting Stage	Mature Fruiting Stage
**Gram-positive**					
*Bacillus cereus*	ATCC 14579	12 ± 0.00 ^a^	12 ± 0.57 ^a^	9 ± 0.57 ^a^	10 ± 1.00 ^a^
*Staphylococcus aureus*	ATCC 25923	-	-	-	-
*Staphylococcus epidermidis*	CIP 106510	-	-	-	-
*Listeria monocytogenes*	ATCC 19115	9 ± 0.57 ^d^	8 ± 0.57 ^c^	-	7 ± 0.00 ^d^
*Enterococcus faecalis*	ATCC 29212	10 ± 0.57 ^c^	9 ± 1.15 ^b^	8 ± 0.00 ^b^	-
*Micrococcus luteus*	NCIMB 8166	9 ± 0.57 ^d^	9 ± 0.57 ^b^	9 ± 0.57 ^a^	9 ± 0.57 ^b^
**Gram-negative**					
*Escherichia coli*	ATCC 85218	-	-	-	-
*Pseudomonas aeruginosa*	ATCC 27853	-	-	-	-
*Salmonella typhimurium*	ATCC 1408	-	-	-	-
*Aeromonas hydrophila*	ATCC 7566	9 ± 0.00 ^d^	8 ± 0.57 ^c^	9 ± 0.57 ^a^	9 ± 0.00 ^b^
*Vibrio vulnificus*	ATCC 27962T	11 ± 1.15 ^b^	9 ± 0.57 ^b^	-	-
*Vibrio alginolyticus*	ATCC 33787	9 ± 0.57 ^d^	9 ± 0.57 ^b^	8 ± 0.57 ^b^	8 ± 0.00 ^c^
*Vibrio cholerae*	non-O_1_ IPT	9 ± 0.00 ^d^	8 ± 1.00 ^c^	-	-
*Vibrio parahaemolyticus*	ATCC 17802	-	-	-	-

Inhibition zone (IZ) calculated as diameter around the disc (mm). The diameter of disc was 6 mm. No antimicrobial activity (–), inhibition zone < 1 mm. Weak inhibition zone, inhibition zone 1 mm. Slight antimicrobial activity, inhibition zone 2 to 3 mm. Moderate antimicrobial activity, inhibition zone 4 to 5 mm. High antimicrobial activity, inhibition zone 6 to 9 mm. Strong antimicrobial activity, inhibition zone > 9 mm. Mean values ± S.D.; Statistical analysis was performed by one-way ANOVA, followed by Duncan test. In each line different letters mean significant differences (*p* < 0.05).
